# Efficacy of Different Combinations of Direct-Acting Antivirals Against Different Hepatitis C Virus-Infected Population Groups: An Experience in Tertiary Care Hospitals in West Bengal, India

**DOI:** 10.3390/v17020269

**Published:** 2025-02-16

**Authors:** Sagnik Bakshi, Partha Chattopadhyay, Mahiuddin Ahammed, Raina Das, Moumita Majumdar, Supradip Dutta, Shreyasi Nath, Anwesha Ghosh, Uttaran Bhattacharjee, Upasana Baskey, Provash Chandra Sadhukhan

**Affiliations:** 1 Indian Council of Medical Research, National Institute for Research in Bacterial Infections P-33, Scheme XM, CIT Road, Beliaghata, Kolkata 700010, West Bengal, India; bakshi445@gmail.com (S.B.); raina2201@gmail.com (R.D.); majumdarmoumita840@gmail.com (M.M.); supradipdutta.sd@gmail.com (S.D.); shreyasiicmr29@gmail.com (S.N.); anwesha.zoology@gmail.com (A.G.); uttaran.ap20@gmail.com (U.B.); bupasana5713@gmail.com (U.B.); 2 College of Medicine & Sagore Dutta Hospital, 578 B.T Road, Kolkata 700058, West Bengal, India; drpartha73@gmail.com; 3 IPGME&R and SSKM Hospital, SSKM Hospital Rd, Bhowanipore, Kolkata 700020, West Bengal, India; skmahiuddinahammed@gmail.com

**Keywords:** direct-acting antivirals (DAAs), genotypes, Hepatitis C virus (HCV), non-responder, sustained virologic response (SVR)

## Abstract

Hepatitis C virus (HCV) is a global public health problem, but advancements in HCV treatment have improved the cure rate. This study evaluated the effectiveness of direct-acting antivirals (DAAs) in HCV-infected patients from May 2021 to April 2023 in collaboration with tertiary care hospitals in West Bengal. The HCV viral load was monitored via qRT-PCR. Sanger sequencing was performed to determine the HCV genotypes. The clinicians prescribed the patient treatment regime. The maximum number of patients in the study population (N = 398) were compensated cirrhosis patients (46.28%). The overall SVR rate of the study population was 94.47%. The decompensated cirrhosis patients experienced the lowest SVR rate (88.89%). The maximum number of patients were prescribed sofosbuvir/daclatasvir (63.77%), and the lowest SVR rate (93.23%) was observed with this treatment regime. In the study population, GT-3 was the predominant (67.43%) circulating genotype, followed by GT-1 and -4. Among 398 patients, 22 (5.53%) were non-responsive to DAA treatment. Out of these 22 non-responder patients, 77.27% (n = 17) were GT-3-infected (3a:10; 3b:07), followed by GT-1 (1c: 04; 1b: 01). Thus, increasing numbers of DAA non-responsive cases among HCV GT-3-infected and decompensated cirrhosis patients may pose serious threats in the future.

## 1. Introduction

Hepatitis C virus (HCV) is a blood-borne positive-sense~9.6 kb RNA virus and a member of the *Hepacivirus* genus of the *Flaviviridae* family [1]. Approximately 170 million people worldwide and 4.7–10.9 million individuals in India are estimated to be infected with HCV [1,2]. According to global data, the prevalence of HCV viremia in India is approximately 0.5% [2]. Approximately 50–80% of acute-phase HCV infections frequently develop into chronic hepatitis, which increases the risk of liver cirrhosis and hepatocellular carcinoma (HCC) [1]. HCV exhibits extensive genetic diversity. It is classified into 8 genotypes (GTs) and 86 subtypes with diverse geographical prevalence [3]. From early 1990 to 2011, pegylated interferon (Peg-IFN) or Peg-IFN with ribavirin (RIB) were considered the gold standard for HCV therapy. However, owing to the low viral clearance response, high cost, and adverse side effects for patients, there is a demand for better and more-effective treatments [4]. In August 2011, first-generation direct-acting antiviral (DAA) drugs were introduced, which remarkably transformed the scenario of HCV treatment [5]. Afterward, the American Association for the Study of Liver Diseases (AASLD) and the Infectious Diseases Society of America (IDSA) published HCV treatment guidelines [6]. However, treatment modifications may be required according to the patients’ treatment responses and comorbidities. In 2018, the Indian government launched the National Viral Hepatitis Control Program (NVHCP) and guidelines for the management of viral hepatitis so that HCV can be eradicated from India by 2030 [7].

In India, the HCV genotype distribution pattern is heterogeneous. Most patients in India’s northern, eastern, and western regions are infected with GT-3, while the maximum number of GT-1 infection cases are reported from southern India [8]. Several studies have reported that GT-3 is comparatively more complex to treat than other genotypes because it is associated with higher chances of complications such as fatty liver disease, fibrosis, and HCC [9]. Thus, it is necessary to understand and evaluate the effectiveness of DAAs in various HCV-infected high-risk groups (HRGs) and the general population in India. The present study aimed to assess the effects of these new-generation drugs on HCV-infected patients. Hence, the present study was conducted to determine the efficacy of DAAs in HCV-infected patients in West Bengal, India.

## 2. Materials and Methods

### 2.1. Study Design

A total of 414 HCV RNA-positive patients were enrolled in this study. We were able to evaluate 398 patients from May 2021 to April 2023. HCV-infected high-risk group (HRG) patients, such as β-thalassemia and chronic kidney disease (CKD) patients and the general population with compensated or decompensated cirrhosis of the liver, were the major participants in this study. Demographic and clinical data were recorded on the prescribed case report form (CRF). All eligible patients were adults (≥18 years old), had follow-up clinical data, and completed the course of treatment. Patients who were less than 18 years old; who were co-infected with hepatitis A, B, E, or human immunodeficiency virus (HIV); who did not complete the course of treatment; or who did not have follow-up clinical data were excluded from this study.

### 2.2. Treatment Regimens

The enrolled patients were treated with sofosbuvir (SOF)/daclatasvir (DCV) ± ribavirin (RIB) or SOF/velpatasvir (VEL) ± RIB. Under the NVHCP guidelines, 400 mg SOF with 60 mg DCV or 400 mg SOF with 100 mg VEL for 12 weeks was prescribed for all patients (except those with decompensated cirrhosis). For patients with decompensated cirrhosis of the liver, treatment with DAAs was continued, and patients were followed up for 24 weeks. The inclusion and dosage of ribavirin (1000 mg or 1200 mg) are based on several factors, such as the HCV genotype, hemoglobin level, body weight (≤75 kg or ≥75 kg), liver cirrhosis status, and renal function conditions [8]

### 2.3. Patient Follow-Up

HCV RNA was measured via a Qiagen real-time qRT-PCR kit (QuantiFast Pathogen RT–PCR + IC kit) based on amplification of the 5′UTR of the HCV genome before initiation and at the end of treatment (12 weeks). Determination of viral RNA before initiation and after the completion of therapy helped to analyze drug efficacy. The baseline limit for viral detection was <50 IU/mL [10].

### 2.4. Amplification of HCV Core Gene and Genotype Determination

The HCV genotype was determined before initiation and at the end of therapy (in the case of non-responder patients). Briefly, viral RNA was extracted from 140 µL of the patients’ serum samples using a QIAamp viral RNA mini kit (Qiagen, Hilden, Germany) per the manufacturer’s protocol and eluted in 50 µL elution buffer. For HCV genotyping, the partial core gene was amplified (405 bp) with genome-specific primers by nested RT-PCR. The primer sequences are listed below in Table 1.

Briefly, in the first round a one-step RT-PCR was performed in 25 µL total reaction volume containing 5 µL HCV RNA. The reaction mixture comprised 0.4 U avian myeloblastosis virus reverse transcriptase (AMV-RT) (Promega, Madison, WI, USA), 0.8 mM dNTPs (Eurogentec, Seraing, Belgium), 5 mM dithiothreitol (DTT), (Sigma-Aldrich, St. Louis, MO, USA), 10 µM core outer forward (CoF1) and reverse primers (CoR1) (IDT, Coralville, IA, USA), 5X Go Taq Flexi buffer (Promega, Madison, WI, USA), 25 mM MgCl_2_ (Promega, Madison, WI, USA), and 0.5 U Go Taq Flexi DNA polymerase (Promega, Madison, WI, USA). The RT-PCR condition was 42 °C for 60 min followed by 94 °C for 5 min followed by 35 cycles of 94 °C for 1 min, 55 °C for 30 s, and 72 °C for 1 min, and the final extension was 72 °C for 5 min. The PCR reaction was performed in the ABI, Veriti 96-well thermal cycler. The second round of nested PCR was performed in 25 µL total reaction volume using 3 µL of the first round’s RT-PCR product. The inner set of primers (CoF2 and CoR2) (Table 1) was used in the second round with all the reagents used in the first round except AMV-RT and DTT. We followed the same nested PCR cycle conditions, only the RT step (42 °C for 60 min) was omitted. The PCR amplicons (405 bp) were electrophoresed in 1.5% agarose gel, stained with ethidium bromide (EtBr), and visualized under a gel documentation system (BioRad, Hercules, CA, USA). Positive PCR amplicons (405 bp) were gel-excised and purified using the Wizard^®^ SV gel and PCR Clean-up System (Promega, Madison, WI, USA) according to the manufacturer’s protocol. The gel-purified products underwent Sanger sequencing using the Big Dye Terminator 3.1 kit (Applied Biosystem, Austin, TX, USA) following the manufacturer’s protocol in an automated DNA sequence 3730 XL (ABI, Van Allen Way, Carlsbad, CA, USA). The sequences were aligned and edited using the Bio Edit tool and genotypes and subtypes were determined by NCBI genotyping tools (www.ncbi.nlm.nih.gov/projects/genotyping/) (accessed on 13 April 2023) [11,12].

### 2.5. Definitions of SVR, Treatment Relapse, and Non-Responders

The patients were categorized into two groups: SVR patients and non-responders. The sustained virologic response (SVR_12_) corresponds to undetectable HCV RNA after treatment completion of 12 weeks, whereas non-responders, defined as HCV RNA, were detectable throughout the treatment span [13].

### 2.6. Statistical Analysis

Continuous variables are expressed as the mean ± standard deviation (SD). For categorical data, the outcome was represented as a percentage. The mean (IQR) was calculated for the clinical data. All the graphs were generated via R version 4.2.2 (R Foundation for Statistical Computing, Vienna, Austria).

## 3. Results

### 3.1. Baseline Characteristics

**Figure 1:**
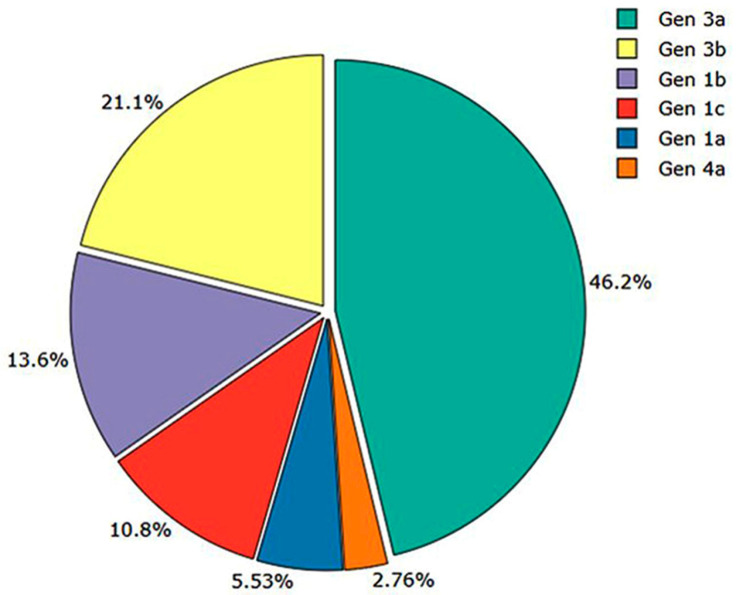
HCV genotype distribution among the study population (N = 398).

The majority of the participants in this study were male (59.55%; n = 237). The mean age of the study population was 37.85 ± 18.76 years. A low DAA efficacy (92.38%) was observed among the 40–50-year-old age group (Table 2). Among the 398 participants, 28.14% (n = 112), 18.09% (n = 72), 46.98% (n = 187), and 6.78% (n = 27) had β-thalassemia, CKD, compensated cirrhosis, and decompensated cirrhosis, respectively (Table 2). The genotype prevalence analysis revealed that GT-3 was the prevalent genotype (67.34%; 3a: 46.23% and 3b: 21.10%), followed by GT-1 (29.90%; 1a: 5.53%, 1b: 13.57% and 1c: 10.80%) and GT-4 (2.76%; 4a: 2.76%) (Table 2 and Figure 1). All the clinical results are listed in Table 3.

### 3.2. Treatment Regime

The most commonly prescribed DAA combination was SOF/DAC (63.07%; n = 251), followed by SOF/VEL (15.58%; n = 62), SOF/DCV + RIB (14.57%; n = 58), and SOF/VEL + RIB (6.78%; n = 27) (Table 4). Overall, 63.43% (170 out of 268) of the GT-3-infected population and 68.06% (81 out of 119) of the GT-1-infected population were treated with SOF/DAC, whereas 63.63% (7 out of 11) of the GT-4-infected population was treated with SOF/VEL (Table 4). Among the high-risk patients, 82.14% (n = 92) of the β-thalassemia patients, 58.33% (n = 42) of the CKD patients, and 58.82% (n = 110) of the compensated cirrhosis patients were prescribed the combination of SOF/DAC, whereas SOF/VEL + RIB was prescribed for most of the decompensated cirrhosis patients (n = 13; 48.15%) (Table 5).

### 3.3. DAA Treatment Response

**Figure 2:**
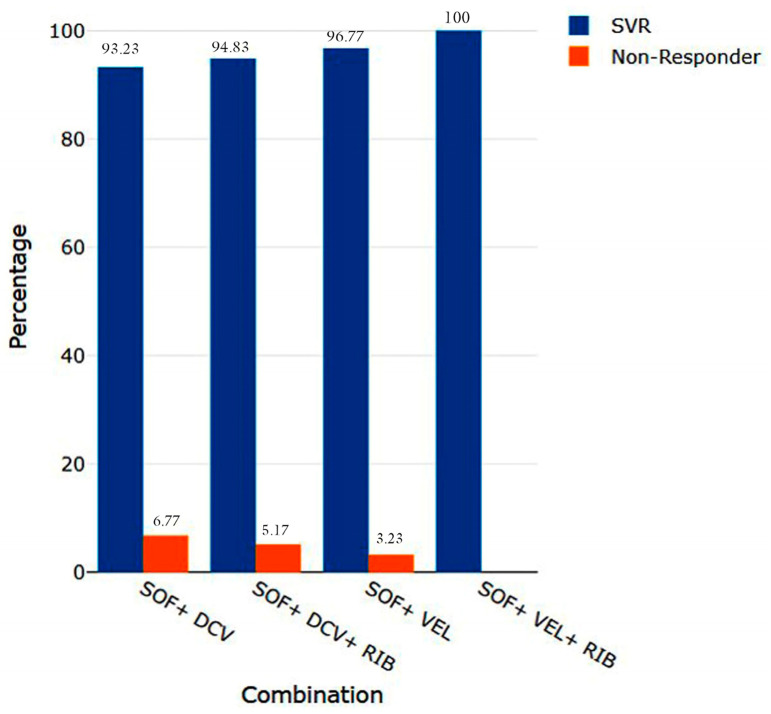
Efficacy of direct-acting antivirals (DAAs) among HCV-infected patients (N = 398).

The overall SVR rate of the study population was 94.47% (n = 376). Briefly, the combination of SOF/DAC ± RIB was able to manage 93.23%-to-94.83% of the SVR, whereas SOF/VEL ± RIB accomplished an SVR rate of 95.16%-to-100% (Figure 2). DAAs with RIB had a better SVR rate (97.65%) than did DAAs without RIB (93.61%) (Table 2). Among the 398 patients, 22 (5.52%; male: 16 and female: 6) were non-responders to DAA treatment (Table 2). Among these 22 patients, 45.45% (n = 10) were in the general population with compensated liver cirrhosis, followed by β-thalassemia (n = 7; 27.27%), CKD (n = 3; 13.64%), and decompensated cirrhosis (n = 3; 13.64%) (Table 2). Patients treated with SOF/DAC (n = 17; 93.23%) had a lower SVR rate than those treated with other combinations (Figure 2). Among the 22 non-responder individuals, 77.27% (n = 17; 3a: 10, 3b:7) were HCV GT-3-infected patients (Table 2).

## 4. Discussion

HCV infection is one of the leading causes of liver damage worldwide. This virus causes approximately 290,000 deaths every year [14]. However, direct-acting antiviral (DAA) therapy against HCV has improved the prognosis and prophylaxis of HCV infection worldwide. In a highly populated country such as India, where HCV GT-3 is the predominant genotype, monitoring the progression of treatment efficacy is crucial because this particular genotype is comparatively more challenging to treat than other genotypes. This study focuses on determining the DAA efficacy in eliminating HCV.

The efficacy of DAAs among different HCV-infected age groups, sexes, populations, and genotypes in India has yet to be reported well since the launch of the NVHCP in 2018. Data from this study revealed that the participation of males was much greater (59.55%) than that of females (35.45%) (Table 2). The low participation of females may be due to a lack of awareness of and negligence in their health, which is still evident in some regions of India. However, the DAA efficacy rate was promising among females (96.27%) compared with males (93.25%) (Table 2). These data corroborate those of a study conducted in Egypt [15]. However, more research needs to be conducted on the potential cause of the better efficacy of DAAs among females. The age-wise study revealed a low DAA efficacy (92.38%) among the 40–50 age group. Additionally, 18–28-year-olds (93.42%) and 51–61-year-olds (93.90%) were not able to achieve satisfactory SVR rates (Table 2). This may be because most of the general population with compensated cirrhosis, β-thalassemia, and decompensated cirrhosis non-responder patients belong to these groups. The evaluation of the efficacy of DAAs among different HCV genotypes was a critical target of this study. Three GT-1 subtypes (GT-1a, -1b, and -1c), two GT-3 subtypes (GT-3a and -3b), and one GT-4 subtype (GT-4a) were circulating in this study population (Figure 1). GT-1a- and -1b-infected patients achieved 100% and 98.15% SVR, respectively. However, GT-1c (90.7%) had a lower clearance rate than the other GT-1 subtypes (Table 2). In this geographical region, the incidence of HCV GT-1c (~57%) infection is relatively high among CKD patients. Among the four GT-1c non-responders, three patients had kidney problems. The lower attainment of SVR among patients with GT-1c infection raises severe concerns for CKD patients. Regarding the SVR rate assessment against GT-3, the SVR rates of DAAs against GT-3a and -3b were approximately 94.56% and 91.66%, respectively (Table 2). A total of 22 patients were non-responders; a maximum number of GT-3-infected patients (n = 17; 3a: 10, 3b: 07) were non-responsive to DAA therapy (Table 2). Although the number of GT-3a (n = 10) non-responder cases was high, the overall SVR achievement rate for GT-3b (91.66%) was low compared with that for GT-3a (Table 2). Several Asian and European studies reported the underperformance of DAAs against HCV GT-3b. This study’s findings validated those of previous reports and our previous work [16,17,18]. Moreover, the emergence of genotype-specific resistance-associated and baseline mutations in the viral genome of the DAA binding site (NS5A and NS5B) may be a possible reason for these non-responder cases [17,18]. The SVR rate achieved against GT-4 was 100%, suggesting that DAA therapy is effective for GT-4-infected patients (Table 2). The DAA SVR rate among different HCV subtypes was significant (95.8%, *p* ≤ 0.0001, CI: 90.31–101.4). Nevertheless, the low efficacy rate among GT-1c- and -3b-infected patients may be a serious concern in the future in this region.

Another major highlight of this study was the assessment of the efficacy of DAAs among different population groups. The overall efficacy of DAAs within the study population was 94.47%, which is lower than that reported in Saudi Arabian (97.33%), Chinese (97.56%), Taiwanese (97.95%), Japanese (99%), Russian (99%), and Brazilian (96%) populations [19,20,21,22,23,24]. Moreover, the efficacy rate in our study population was even lower than the Cameroonian (96.2%) and Egyptian (95.23%) populations [25,26]. However, the overall efficacy rate was better than the Canadian (93%), German (92%), British (91%), and Italian (88.2%) populations [27,28,29,30]. Additionally, we observed that the efficacy of DAAs in our study population was similar to the US population (94%) [31]. The SVR rates achieved by β-thalassemia, CKD, and the general population with compensated cirrhosis and decompensated cirrhosis patients were 94.65% (106/112), 95.83% (69/72), 94.65% (177/187), and 88.89% (24/27), respectively (Table 2). Several clinical studies reported that the efficacy of DAAs in compensated cirrhosis patients ranged between 92% and 96% [14]. Moreover, Verna et al. reported that the overall efficacy of DAA treatment among decompensated cirrhosis patients was 90.5% [32]. Therefore, these study data substantiate previous reports on the sluggishness and underperformance of DAA efficacy in decompensated cirrhosis patients and indicate severe concerns for the future. The possible reasons for this result are that most β-thalassemia patients, and the general population with compensated cirrhosis and decompensated cirrhosis non-responder patients, are infected with GT-3. Compared with other genotypes, GT-3 is associated with a high risk of complications such as fatty liver disease, HCC, and fibrosis, making it more complicated to treat. Singh et al. reported that the prevalence and chance of progression of cirrhosis are greater in GT-3 than in other genotypes. The persistence of HCV GT-3 infection could lead to the progression of HCC and liver failure, especially among non-responder cirrhosis patients and decompensated cirrhosis patients, which results in mortality [33]. This study revealed a connection between lower SVR rates and GT-3-infected β-thalassemia, cirrhosis, and decompensated cirrhosis patients. In addition, the SVR efficacy of various combinations of prescribed DAA drugs was explored, and the overall efficacy of the DAAs was significant (*p* ≤ 0.0001). The SVR rates achieved by SOF/DCV, SOF/DCV + RIB, SOF/VEL, and SOF/VEL + RIB were 93.23%, 94.83%, 96.77%, and 100%, respectively (Figure 2). These data suggest that the inclusion of ribavirin increases the efficacy of both SOF/DAC and SOF/VEL treatment combinations. The low SVR rate of SOF/DCV± RIB may be due to the emergence of most GT-3 non-responders (14 out of 17) with these two combinations. Therefore, SOF/VEL± RIB may be prescribed as an alternative treatment for non-responder patients. The data indicate that the treatment of GT-3 remains a concern in the post-DAA era.

Certain inherent limitations exist in this study. GT-3 was the prevalent genotype followed by GT-1 and -4 in the study population (Figure 1). Moreover, the maximum non-responder cases were observed in GT-3 followed by GT-1 and most of the HCV-infected patients showed non-responsiveness against SOF/DCV (Figure 2). Several studies reported that genotype-specific resistance-associated substitutions (RASs) have an impact on the SOF/DCV efficacy in the HCV-infected patient population. Dietz et al. mentioned that specific RASs like Q30H/R, L31M, and Y93H are associated with DCV failure among HCV-infected GT-1 and -3 patients and the NS5B-specific RAS S282T is linked with GT-3-infected SOF failure patients [34]. Di Stefano et al. stated the circulation of the NS3-specific RAS V170I and NS5A-specific RASs like L28M, L30R, and L28 + M31L in the SOF/DCV-treated HCV-infected GT-4 population [35]. The major drawback of this study was that baseline NS5A/NS5B RASs were not mentioned, and RAS data would help minimize this study’s drawback. However, this group is working on an RAS analysis study. Moreover, this study started during the COVID-19 period. Thus, this study faced the challenge of maintaining patients’ details and proper connections with them during the initial period, and it failed to channel more patients to participate because of the lockdown. However, improving the pandemic made it possible to channel contact information correctly. Another drawback of this study was that the evaluation of DAA efficacy among HCV/HIV-co-infected patients was not performed because of a scarcity of data concerning this group of co-infected patients. Assessment of DAA efficacy among this particular group of patients would lessen the issue with the literature.

## 5. Conclusions

A detailed scenario of the efficacy of DAAs against HCV infection in West Bengal, India, is illustrated. The SVR rate was sluggish against decompensated cirrhosis and kidney failure patients. The DAA efficiency against GT-1a and -1b was promising, but the lower DAA efficacy against GT-3 (3a and 3b) and GT-1c may be a concerning issue for the community. Overall, the SVR rate among the Indian population was lower than in other countries. This study highlights the ongoing challenge of eliminating HCV by 2030 in India. Therefore, an effective HCV vaccine is immediately needed to relieve the burden of this disease.

## Figures and Tables

**Table 1:** List of primers used for partial core amplification.

Primer Name	Primer Sequences (5′ to 3′)
CoF1	ACTGCCTGATAGGGTGCTTGC
CoR1	ATGTACCCCATGAGGTCGGC
CoF2	AGGTCTCGTAGACCGTGCA
CoR2	CACGTTAGGGTATCGATGAC

**Table 2:** Demographic distribution of the study population (N = 398).

	Total Population (N = 398)	SVR (n = 376)	Non-Responder (n = 22)
Age (Years) (Mean ± SD)	37.85 ± 18.76	37.85 ± 18.74	38.01 ± 18.06
Age Group			
18–28	76 (19.09%)	71 (93.42%)	5 (6.58%)
29–39	77 (19.34%)	75 (97.40%)	2 (2.60%)
40–50	105 (26.38%)	97 (92.38%)	8 (7.62%)
51–61	82 (20.60%)	77 (93.90%)	5 (6.1%)
62–72	58 (14.57%)	56 (96.55%)	2 (3.45%)
Gender			
Male	237 (59.55%)	221 (93.25%)	16 (6.75%)
Female	161 (35.45%)	155 (96.27%)	6 (3.73%)
High-Risk Group (HRG) populations			
β-thalassemia	112 (28.14%)	106 (94.65%)	6 (5.35%)
Chronic Kidney Disease	72 (18.09%)	69 (95.83%)	3 (4.17%)
General population with compensated cirrhosis and decompensated cirrhosis			
Compensated cirrhosis	187 (46.98%)	177 (94.65%)	10 (5.34%)
Decompensated cirrhosis	27 (6.78%)	24 (88.89%)	3 (11.11%)
Genotypes			
Genotype 1a	22	22 (100%)	0 (0.00%)
Genotype 1b	54	53 (98.15%)	1 (1.85%)
Genotype 1c	43	39 (90.7%)	4 (9.3%)
Genotype 3a	184	174 (94.56%)	10 (5.43%)
Genotype 3b	84	77 (91.66%)	7 (8.34%)
Genotype 4a	11	11 (100%)	0 (0.00%)
DAA Therapy			
With RIB	85 (21.35%)	83 (97.65%)	2 (2.35%)
Without RIB	313 (78.64%)	293 (93.61%)	20 (6.39%)

**Table 3:** Clinical marker characteristics among the different study population groups (N = 398).

Clinical Markers	Patient Category			
	β-Thalassemia (n = 112)	Chronic Kidney Disease (n = 72)	Compensated Cirrhosis (n = 187)	Decompensated Cirrhosis (n = 27)
Albumin (g/dL)	4.05 (3.9–4.35)	3.9 (3.9–4.1)	3.65 (3.2–4.05)	2.9 (2.7–3.2)
Globulin	3 (2.9–3.3)	3.1 (2.85–3.55)	3.6 (3.2–4)	3.9 (2.26–5.6)
SGOT (U/L)	60 (55.25–67)	34 (21.8–58.5)	57.5 (44–91.75)	71 (40–98)
SGPT (U/L)	59 (44–74.5)	43 (35.3–52.5)	53 (32.7–72.5)	39 (27–65.75)
Creatinine (mg/dL)	0.7 (0.58–0.8)	9.13 (6.95–14.01)	0.85 (0.7–1)	0.75 (0.63–0.89)
Hemoglobin (g/dL)	6.35 (5.63–7.35)	9.15 (8.1–10.7)	11.3 (8.9–13.1)	8.95 (7.9–10.43)
Total Protein	7.15 (6.8–7.75)	7.05 (6.8–7.28)	7.4 (7–7.75)	6.1 (5.7–7.18)
Bilirubin (Total) (mg/dL)	1.55 (1.4–2.23)	0.65 (0.58–0.92)	1.2 (0.8–1.7)	2 (1.01–3.28)
Urea (mg/dL)	20.5 (18.75–22)	134.3 (93.75–155.5)	23 (19–29)	17.4 (14.5–29.25)
ALP (U/L)	237 (197.5–298.75)	132.2 (123.25–172)	125 (97.36–178)	164.5 (107.5–197.25)

**Table 4:** DAA combinations prescribed among various HCV genotypes in the study population (N = 398).

Combination of DAAs (N = 398)	Gen-1a (n = 22)	Gen-1b (n = 54)	Gen-1c (n = 43)	Gen-3a (n = 184)	Gen-3b (n = 84)	Gen-4a (n = 11)
SOF + DCV	90.90% (n = 20)	57.41% (n = 31)	69.77% (n = 30)	73.36% (n = 135)	41.66% (n = 35)	NIL
SOF + DCV + RIB	4.54% (n = 1)	14.81% (n = 8)	27.91% (n = 12)	8.15% (n = 15)	25% (n = 21)	9.09% (n = 1)
SOF + VEL	4.54% (n = 1)	20.37% (n = 11)	2.32% (n = 1)	10.87% (n = 20)	26.48% (n = 22)	63.63% (n = 7)
SOF + VEL + RIB	NIL	7.41% (n = 4)	NIL	7.61% (n = 14)	7.14% (n = 6)	27.27% (n = 3)

SOF = Sofosbuvir, DCV= Daclatasvir, VEL = Velpatasvir, RIB = Ribavirin, DAAs = Direct-acting antivirals, Gen = Genotype.

**Table 5:** DAA combinations prescribed among various HCV study populations (N = 398).

Combination of DAAs (N = 398)	Patient Category (n = 398)			
	β-Thalassemia (n = 112)	Chronic Kidney Disease (n = 72)	Compensated Cirrhosis (n = 187)	Decompensated Cirrhosis (n = 27)
SOF + DCV	92 (82.14%)	42 (58.33%)	110 (58.82%)	7 (25.92%)
SOF + DCV + RIB	5 (4.46%)	18 (25%)	38 (20.32%)	3 (11.11%)
SOF + VEL	8 (7.14%)	12 (16.66%)	32 (17.11%)	4 (14.81%)
SOF + VEL + RIB	7 (6.25%)	NIL	7 (3.74%)	13 (48.15%)

SOF = Sofosbuvir, DCV = Daclatasvir, VEL = Velpatasvir, DAAs = Direct-acting antivirals, RIB = Ribavirin.

## Data Availability

The raw data supporting the conclusions of this article will be made available by the corresponding author upon request.
